# The Influence of Flexible Employment on Workers’ Wellbeing: Evidence From Chinese General Social Survey

**DOI:** 10.3389/fpsyg.2022.771598

**Published:** 2022-02-25

**Authors:** Teng Liu, Qian Liu, Daokui Jiang

**Affiliations:** ^1^Business School, Shandong Normal University, Jinan, China; ^2^School of Economics and Management, Guangdong Construct Polytech, Guangzhou, China

**Keywords:** flexible employment, workers’ wellbeing, labor income, social insurance, social survey

## Abstract

Based on the 2017 China General Social Survey data, with 5,439 observations as research objects, this paper empirically tests the impact of flexible employment on workers’ wellbeing and introduces labor income as mediator and social security as moderator to explore the mechanism of action. The empirical results show that: flexible employment has an inverted U-shaped relationship with workers’ wellbeing, which indicates that increasing employments’ flexibility will first rise and then reduce their perceived subjective wellbeing after reaching the peak; labor income plays a mediating role in the relationship of flexible employment and wellbeing of workers; social security moderates the mediating effect of labor income whereas the moderating role in the relationship between flexible employment and workers’ wellbeing is not observed. Implications and future development of flexible employment are discussed.

## Introduction

“Flexible employment” originally came from the “informal employment” proposed by the International Labor Organization (ILO, 1972), which refers to “the form of employment in the informal sector.” In 2003, the ILO re-evaluated this concept and included those who were informally employed in the formal sector as “flexible employment.” In the process of talent development and labor market transformation, flexible employees, as a special group that has received wide attention, have shown a surge in their number in recent years. The National Bureau of Statistics stated in a press conference that the scale of flexible employment increased by about 20% in 2020, and it will have great growth potential in 2021. Under the normalization of epidemic prevention and control, the form of flexible employment undoubtedly provides a boost to stable employment (Spurk and Straub, 2020). The “Opinions on Supporting Multi-channel Flexible Employment” issued by State Council of China clearly pointed out that supporting flexible employment should be an important measure to stabilize employment and ensure the employment of residents, encourage the development of self-employed businesses, increase part-time employment opportunities, and support the development of new employment patterns. This guiding opinion affirms the important significance of flexible employment for social and economic development from the national level.

In the past, flexible employment mainly included traditional labor dispatch, temporary employment, etc. With the continuous deepening and application of Internet technology in various industries, the platform economy is booming, and new employment forms have emerged (He et al., 2019). Some professional and technical talents with attainments in a certain field, highly educated or knowledgeable intellectuals are attracted by “work flexibility” and join the ranks of freelancers. The scale of the flexible labor market continues to expand. In addition, companies are also concerned about this form of employment. During the epidemic, flexible working systems and flexible employment models emerged (Vossemer et al., 2018; Amit and Karen, 2020), such as “shared employees.” Innovative employment models have solved the “urgency” of enterprise employment and have caused many companies to follow suit. Therefore, in the context of the expansion of the scope of flexible employment, the diversification of forms, and the surge in the number of employees, it is meaningful to explore the happiness level of flexible employment.

Easterlin first explored happiness from the perspective of economics (Oishi and Kesebir, 2015), and then, the research on the factors affecting happiness has become more abundant. Studies have shown that the level of economic development, inflation, government public services, green space (Zhao et al., 2019), social capital, and geographic context (Clark et al., 2019) have varying degrees of impact on happiness. In addition, the quality of employment is also an important variable explaining happiness (Aerden et al., 2015). Work is an important part of people’s lives; therefore, labor remuneration, job training, and safety guarantee may affect the level of happiness. However, scholars have different views on the relationship between flexible employment and happiness. Some suggest that flexible employment can increase the level of happiness of workers. Flexible employment tends to have higher autonomy and selection on work time or work place (Okulicz-Kozaryn and Golden, 2018). For individuals who cannot find a satisfactory job within a short period of time, flexible employment can help them obtain certain economic compensation during the “transition period” (Guest, 2004). Another more widely accepted view is that flexible employment will reduce workers’ wellbeing. Green and Leeves (2013) confirmed the existence of a correlation between job insecurity and flexible employment, and job insecurity affects individual wellbeing and organizational performance. It is believed that the insecurity caused by job instability has a greater impact on happiness than the pleasure brought by job flexibility.

Does flexible employment increase or decrease happiness? How does flexible employment affect happiness? These questions are worth thinking about. Existing research rarely discusses that. Although some scholars have proposed the impact of employment on happiness from different aspects (Angrave and Charlwood, 2015; Kirsten et al., 2016), there is insufficient research on the impact of flexible employment on happiness, and lacking in-depth discussion of the internal mechanism of the two as well. This study uses the 2017 Chinese General Social Survey (CGSS) data to reveal the impact of flexible employment on workers’ wellbeing, and provides marginal contributions: First, it introduces quadratic term of flexible employment to explore the non-linear relationship between flexible employment and laborers’ wellbeing and construct theoretical models to explain the two seemingly contradictory conclusions put forward by the above-mentioned different scholars; the second is to incorporate labor income and social security as mediating variable and moderating variable into the research model and further explore the effect of flexible employment on wellbeing to provide more explanations and evidence for this study.

## Literature Review and Hypotheses

### Flexible Employment and Workers’ Wellbeing

Flexible employment includes part-time employment, temporary employment, dispatched labor, part-time labor, self-employed labor, network platform shared labor, etc. (Razumov et al., 2021). Scholars define flexible employment mainly around complex/informal labor relations and work. Flexible time and uncertain workplaces (Xue et al., 2014) reflect the dual characteristics of “informality” and “flexibility” of this form of employment. Therefore, we define “flexible employment” as “informal employment with flexible working time and flexible workplace,” which takes freelancing into account instead of remote work or working from home in formal employment. Employees formally employed by enterprises or organizations need to accept different types of control from the organization, while flexible employment employees can “escape” organizational control to a certain extent and achieve more self-control. Flexible employment breaks the time and space limitations in the traditional employment system, provides more options for workers, especially low-skilled people, and solves the short-term employment problem of some people (Rubery et al., 2016). Flexible jobs provide more flexibility for workers, so they value job flexibility and even are willing to pay for it (He et al., 2021). For high-skilled people, flexible labor relations meet their time flexibility needs, help them to effectively exert their comparative advantages under flexible working conditions, achieve greater personal benefits, and improve job satisfaction (Green and Heywood, 2011).

Nevertheless, from a broader perspective, the negative problems cannot be underestimated. According to the theory of dual segmentation of the labor market, workers in the primary labor market can generally enjoy a better working environment, higher self-employment benefits, and more stable jobs, while those in the secondary labor market often face worse work and poor life quality, and their welfare and health conditions are also worse (Kompier et al., 2009). In fact, more workers engaged in flexible employment gather in the secondary labor market. Firstly, flexible employees usually do not sign a formal labor contract with the employer, and there is a higher risk of being dismissed and a greater probability of facing unemployment. The high unemployment rate especially has an adverse effect on temporary employees (Aleksandra et al., 2020). Secondly, the flexible labor market is developing rapidly, but market supervision has not kept up. The relevant systems are not yet complete, and the legitimate rights and interests of more flexible employees cannot be protected, such as lack of social security, poor working environment, and long working hours. There are relatively few opportunities for them to receive job training and improve human capital (Benach et al., 2016). The lack of legitimate rights greatly reduces their level of happiness. Thirdly, compared with regular employees, flexible employees have blurred work–family boundaries, so they face more pressure from work and family, and contradictions are more prominent (Golden et al., 2006). In particular, flexible employment itself has the characteristics of “work more, get more money.” “Go wherever you have a job” may encroach on time outside of your original work, resulting in “work and family mistakes.” These different sources of stress will reduce the happiness level of flexible employees. Based on this, put forward the hypothesis:

*H1: Flexible employment has an inverted U-shaped relationship with workers’ wellbeing: increasing employments’ flexibility will first rise and then reduce their perceived subjective wellbeing at the individual level after reaching the peak.*

### The Mediating Role of Labor Income

The field of economics usually substitutes “utility” for “happiness.” It is believed that when income increases, an individual’s disposable budget increases and personal utility increases as a result. This implies a greater sense of happiness. Therefore, mainstream economics supports income is positively related to happiness. Easterlin (2001), as a representative supporter of “relative income determining happiness,” argue that different income groups will change their original material expectations with increasing income. The expectations of people expand as their incomes increase, thereby reducing happiness. This view is also known as the “Easterlin Paradox,” which has caused many scholars to discuss. Research finds that financial problems are more strongly associated with poor wellbeing for the self-employed compared to the wage-employed and low-wage has negative influence on workers’ wellbeing both for self-employees and wage employees (Jenny et al., 2021). Based on CGSS, Ding confirms that both absolute and relative income are positively and significantly correlated with happiness (Ding et al., 2021), but changes in relative income have larger effects on happiness than do changes in absolute income (Ball and Chernova, 2008).

Flexible employment can increase workers’ income. On the one hand, unemployed workers can obtain temporary job as “stepping stone” through flexible employment, thereby increasing their income during the unemployment period. Especially women who are mothers can benefit from flexible working time arrangements and can obtain higher job satisfaction and happiness in life (Xiang et al., 2021). On the other hand, getting multiple jobs increase workers’ total personal labor income. However, according to the relative income theory, people’s perception of individual utility depends not only on absolute income and self-consumption, but also by the income level of others (the reference group), that is, income gap. Some scholars have found that the wage level of flexible employment is generally lower than that of formal employees. First, the labor price in the flexible employment market is at a lower level, and the second is that low-skilled people still account for a higher proportion of flexible employment groups (Huang et al., 2020). Burgoon and Dekker (2010) find that flexible employment generates various kinds of economic insecurity for workers based on individual-level survey data from 15 EU member states. There are noticeable differences in income levels that different groups of workers and those who have formal work have higher incomes than those with informal work on average. Howell (2013) also reached similar conclusions in follow-up studies. Unstable income will reduce the total income level of employees and at the same time increase work insecurity, thereby reducing happiness.

Existing literature studies have basically confirmed the positive correlation between income and happiness, that is, happiness increases as income level increases (Killingsworth, 2021) and decreases with the increase of income gap. Base on the literature reviews above, we propose the following hypothesis:

*H2: Labor income will mediate the association between flexible employment and workers’ wellbeing. That is, flexible employment will negatively impact labor income, which in turn will decrease workers’ wellbeing.*

### The Moderating Role of Social Security

Social insurance is the core content of state social security system. It helps to improve people’s livelihood and maintain social equity. It is of great significance to guarantee life quality of people and enhance the national sense of security. In a large survey of American, individuals with health insurance were more likely to be “very satisfied” or “satisfied” with life (Tran et al., 2017). A large number of existing literature show that there is a positive correlation between pension and subjective wellbeing, which also suggests that pension policies can effectively improve residents’ wellbeing (Fang and Sakellariou, 2016).

The social security status of flexible employees is mostly at a low level (Lippmann and Brown, 2016). On the one hand, flexible employees have no stable employers and lack job protection. Therefore, there is no employers to pay social insurance for them. Individuals decides whether to pay or not at their own expense. However, factors, such as job mobility and information missing, make this independent choice uncertain. Adverse selection appears in the behavior of participating in insurance (Muttaqien et al., 2021). On the other hand, paying social insurance will have a crowding-out effect on the income of workers (Anand, 2017), resulting in loss of established welfare, thereby reducing their willingness to pay. If the worker pays social insurance on time, the medical security and pension will help eliminate some insecurities. Therefore, raising the level of social security can effectively improve the social welfare of flexible employees and enhance their sense of happiness. Based on the views above, we propose the following hypothesis:

*H3a: The direct and/or indirect associations between flexible employment and workers’ wellbeing via labor income will vary as a function of social insurance. The higher level of social security will attenuate the negative impact of flexible employment on workers’ wellbeing.*

With the continuous increase in the number of flexible employment groups, the state has paid more and more attention to the insurance of flexible employees and has also issued relevant policies to support it, aiming to adjust the income gap between different groups through the social insurance system and improve people’s happiness. He and Sato (2013) propose in their research that social security can increase the income of low-income groups and reduce the relative poverty rate, which to a certain extent narrows the income gap. Lu et al. (2020) finds that Urban Residents Basic Medical Insurance and Urban Employees Basic Medical Insurance (UEBMI) have significant increased monthly net income of agricultural migrants, and the income-increasing effect of UEBMI is most obvious in the low-income group. In addition, as an effective means to protect the health of residents, medical insurance plays a significant role in the payment of medical expenses after the insured suffers from illness and alleviates the economic crisis. It can provide certain financial compensation to the insured and reduce the negative impact of health status on wellbeing. Based on empirical evidence, we propose the following hypothesis:

*H3b: Social security will moderate the mediating role of labor income in association between flexible employment and wellbeing. The higher level of social security will attenuate mediating effect of labor income in association between flexible employment and wellbeing.*

**Figure 1:**
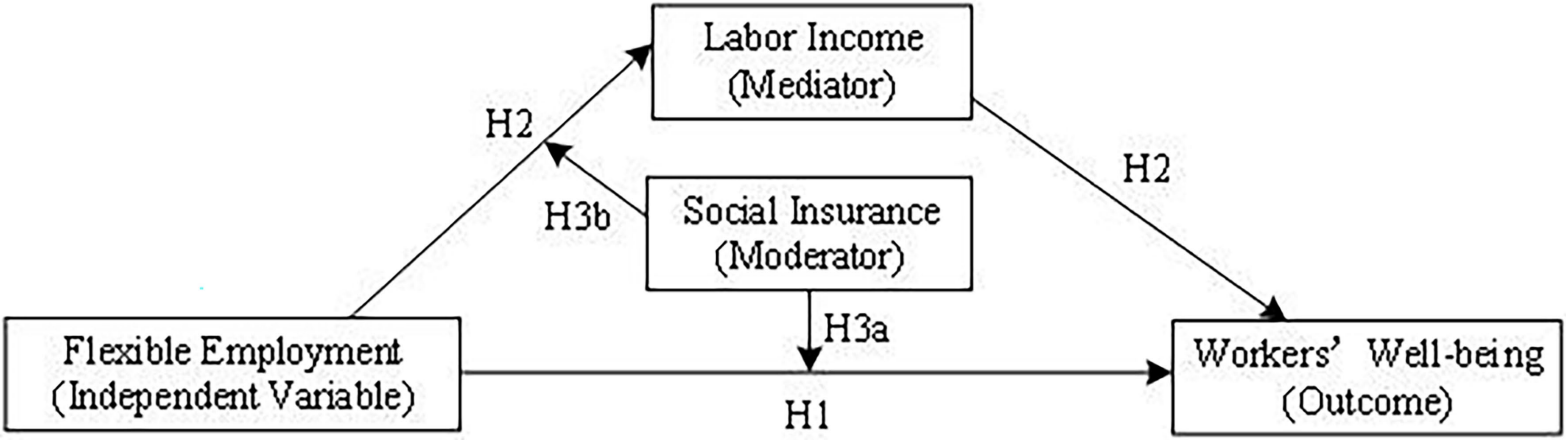
The proposed mediated moderation model.

To sum up, the research model is as follows (Figure 1).

## Materials and Methods

### Sample

The research data comes from the 2017 China Comprehensive Social Survey. The survey, investigating more than 10,000 households across the country every year to provide data for social research, is a national, comprehensive, and continuous large-scale social project jointly implemented by Renmin University of China in 2003 and academic institutions across the country. In 2017, the data sampling design adopted a multi-stage stratified probability home visit method, and a total of 12,582 valid observations were completed, and the data contained 783 variables. The research selects 10 items in the questionnaire for data processing, deletes observations with missing data, data errors, and data with different values. The study obtains a benchmark sample containing 5,439 observations finally.

### Variables

According to the definition and description of variables, this study summarized and sorted out the relevant items in the questionnaire and re-coded the variables, as shown in Table 1 for further research.

**Table 1:** Variable definition and description.

Variable type	Variable name	Variable description
Dependent variable	Flexible employment	Respondent’s degree of flexibility in employment: very inflexible = 1, relatively inflexible = 2, generally flexible = 3, relatively flexible = 4, very flexible = 5
Independent variable	Workers’ wellbeing	Respondents’ perception of happiness in life: very unhappy = 1, relatively unhappy = 2, not happy or unhappy = 3, relatively happy = 4, and very happy = 5
Moderating variable	Social insurance	Respondents’ level of social insurance contributions: very low = 1 (not participating in any social security projects), relatively low = 2 (only participating in 1 social security project), normal level = 3 (participating in 2 social security projects), Relatively high = 4 (participating in 3 social security projects), and very high = 5 (participating in 4 social security projects)
Mediating variable	Labor income	The logarithm of the interviewee’s annual labor income
Control variables	Age	Respondent’s age
	Gender	Male = 1, female = 2
	Education	Elementary school and below = 1, junior high school = 2, high school/secondary school = 3, junior college = 4, undergraduate, and above = 5
	Census register	City = 1, Countryside = 2
	Health	Very unhealthy = 1, relatively unhealthy = 2, general = 3, relatively healthy = 4, very healthy = 5

Flexible employment. We used the question “Which of the following situations is more suitable for your current work situation” to measure “flexible employment” with 5 items. Participants choose form “self-employed entrepreneurs,” “self-employed households,” “formal employed,” “gig employment,” “labor dispatching,” and “freelancing,” where “formal employed” =1, “labor dispatching” =2, “self-employed entrepreneurs” or “self-employed households” = 3, “gig employment” = 4, and “freelancing” = 5. Larger value indicates more flexibility. In order to make the measurement more robust, we tested it with another item which can reflect job flexibility (“To what extent can you decide your own way of working in your current job”). Results show that “flexible employment” significantly positively predicts job flexibility (*b* = .378, *p* < .001), indicating this measurement is reasonable.

Workers’ wellbeing. Regarding the measurement of happiness variables, a large body of literature adopt the self-reporting method, that is, directly taking the respondents’ choices on related questions as the measurement results. With reference to other researchers (Asadullah et al., 2018) in the CGSS data analysis for measuring wellbeing. This study chooses the item “Generally speaking, how do you personally feel about your life?” to measure the explained variable, and the respondent choose from “very unhappy = 1, relatively unhappy = 2, not happy or unhappy = 3, relatively happy = 4, very happy = 5.” Considering the fact that the object of this research is laborers and the data are screened based on this, the outcome variable is named “worker’s wellbeing.”

Social security. Social insurance in CGSS data is measured by using responses to the question, “Are you currently participating in the following social security projects?” on a scale of 1 to 4 where 1 is Urban Basic Medical Insurance/New Rural Cooperative Medical Insurance/Public Medical; 2 = Urban/Rural Basic Pension Insurance; 3 = Commercial Medical Insurance; 4 is Commercial Pension Insurance. Insurance can improve personal wellbeing (Pontarollo et al., 2020), so we suggest that higher level of social security can increase their sense of happiness. Therefore, this study uses the number of social security projects that respondents participate in to measure the level of social security.

Labor income. Using the question “What is your personal occupation/labor income last year (2016)?” to measure labor income and take the logarithm as the variable value. Choosing “annual labor income” instead of “monthly income” can avoid data errors caused by unstable monthly income of flexible employees and more accurately measure the relationship between variables.

Control variables. Drawing lessons from existing studies, age, gender, education level, household registration, and health status may affect the results of the analysis, so these variables are controlled.

## Results

### Descriptive Statistical Analysis

The results of the Pearson correlation coefficients are shown in Table 2. The correlation coefficients between flexible employment and the workers’ wellbeing are positive significantly (*r* = .036, *p* < .001); the correlation coefficients between flexible employment and the labor income, social insurance variables are .212, −.028, respectively and the correlation coefficients between workers’ wellbeing and the labor income, social insurance variables are .172, .130, respectively. The correlation coefficient between core variables and most of the control variables is significant, which indicates the rationality of selecting these control variables, to a certain extent.

**Table 2:** Means, standard deviations, and correlations of the main study variables.

S. No		1	2	3	4	5	6	7	8	9
1.	Gender	1								
2.	Age	−.004	1							
3.	Census register	−.029^*^	.193^***^	1						
4.	Health	.044^**^	−.297^***^	−.173^***^	1					
5.	Education	−.025	−.383^***^	−.431^***^	.238^***^	1				
6.	Flexible employment	−.129^***^	−.102^***^	−.213^***^	.127^***^	.033^*^	1			
7.	Workers’ wellbeing	.030^*^	−.053^***^	−.071^***^	.263^***^	.175^***^	.036^**^	1		
8.	Labor income	−.150^***^	−.203^***^	−.448^***^	.252^***^	.473^***^	.212^***^	.172^***^	1	
9.	Social insurance	.029^*^	.066^***^	−.148^***^	.066^***^	.314^***^	−.028^*^	.130^***^	.261^***^	1
	Mean	1.480	44.540	1.320	3.790	6.200	1.230	3.830	1.306	2.920
	SD	.500	9.997	.468	.983	3.410	1.312	.822	1.250	.890

*N* = 5439.

* *p* < .05;

** *p* < .01;

*** *p* < .001.

### Hypotheses Testing

In this study, the hypothesis test is performed by the hierarchical linear regression method. Gender, age, education level, household registration, and health status, are controlled in the regression model. Prior to the regression analysis, the Variance Inflation Factor (VIF) is checked. The results showed that the VIF value is between 1.055 and 1.715, indicating that there is no serious multicollinearity problem between the variables.

#### Test of Main Effect and Mediating Effect

The results of the level regression analysis of each variable are shown in Table 3. We show the influence of control variables on the wellbeing of workers in Model 4, while we find, in Model 5, the significantly positive effect of FE and significantly negative effect of FE2 on workers’ wellbeing (*b* = .078, *p* < .001; *b* = −.016, *p* < .05) In addition, we tested the value range of the inflection point. Completing with the quadratic function inflection point formula, it can be seen that the inflection point appears near FE = 2.4375, which is in a reasonable range. According to suggestion of Haans et al. (2015), the present results can prove the existence of the inverted U-shaped relationship between flexible employment and workers’ wellbeing. Hypothesis 1 is supported.

**Table 3:** Results of hierarchical regression analysis.

Variables	Labor income			Workers’ wellbeing			
	1	2	3	4	5	6	7
Gender	−.364^***^	−.322^***^	−.267^***^	.076^***^	.081^***^	.094^**^	.117 ^***^
Age	.001	.003^**^	.001	.006^***^	.007^***^	.007^***^	.007^***^
Census register	−.790^***^	−.629^***^	−.502^***^	.049	.072^**^	.089^**^	.133^***^
Health	.156^***^	.126^***^	.097^***^	.214^***^	.209^***^	.252^***^	.244^***^
Education	.116^***^	.114^***^	.078^***^	.038^***^	.037^***^	.038^***^	.031^***^
FE		.480^***^	.472^***^		.078^**^	.111^**^	.070
FE2		−.091^***^	−.366^***^		−.016^*^	−.094^**^	−.062
SI			.125^***^			.068^***^	.057^***^
FE^*^SI			−.010			.027	.027
FE2^*^SI			.004			−.031	−.031
LI							.087^***^
*R* ^2^	.333	.359	.372	.090	.091	.096	.100
Δ*R*^2^	–	.026^***^	.013^***^	–	.001^*^	.004^***^	.005^***^

*N* = 5439, FE, flexible employment, FE2, square of flexible employment, SI, social insurance, LI, labor income.

* *p* < .05;

** *p* < .01;

*** *p* < .001.

According to the method provided by Baron and Kenn to verify the mediating role of labor income between flexible employment and workers’ wellbeing. In Model 2 of Table 3, the effect of flexible employment on labor income is significantly positive (*b* = .480, *p* < .001), and the regression coefficient of the square term of flexible employment negatively predicted labor income (*b* = −.091, *p* < .001). Labor income has a positive and significant impact on workers’ happiness (*b* = .057, *p* < .001), seen in Model 7, indicating that the mediating effect of labor income exists. High labor income stronger the sense of worker’s wellbeing. On the contrary, the lower level of income will reduce the worker’s happiness. Finally, we used bootstrap method to examine the mediating effect of labor income. Results show that the 95% confidence interval of the mediation effect does not include 0 in the test of both FE (95%CI = [.0044, .0102]) and FE2 (95%CI = [.006, .0017]), indicating that the mediation effect of labor income has reached a significant level, which confirms that labor income plays an mediating role between flexible employment and workers’ wellbeing. Hypothesis 2 is supported.

#### Test of Moderating Effect

**Figure 2:**
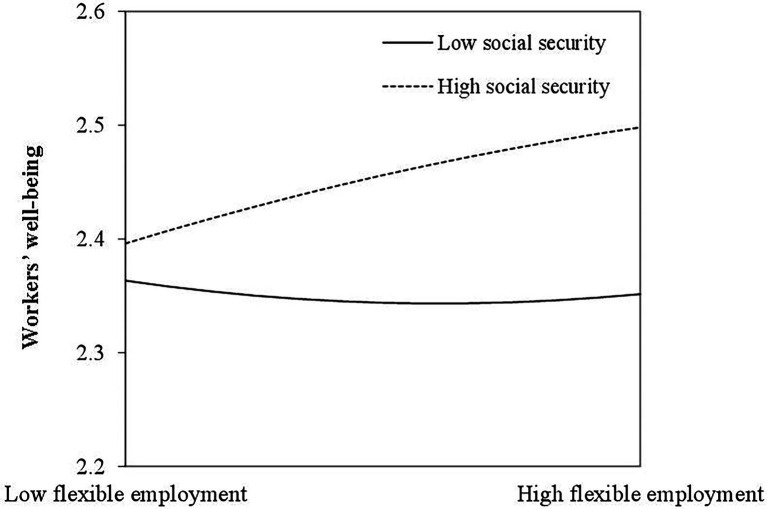
The direct moderating effect of social security.

According to the suggestions of Aiken and West, when the adjustment effect is tested, we make the square term of flexible employment and social security variables standardized and then calculate interaction term to avoid the problem of collinearity. The results of Model 6 (see Table 3) show that social security has a significant positive effect on the wellbeing of workers (*b* = .068, *p* < .001), but the interaction item of FE and SI, positively predict the wellbeing of workers insignificantly as well as the interaction item of FE2 and SI. In order to more intuitively understand the moderating effect of social insurance, we drew a diagram of the moderating effect, as shown in Figure 2. Under low-level social security, flexible employment has an unobvious U-shaped relationship with workers’ wellbeing, that is, with the increase in employment flexibility, employers tend to feel unhappy. However, under the high level of social security, workers’ wellbeing will be improved due to flexible employment, but the effect is not significant. Hypothesis 3a is not supported.

**Figure 3:**
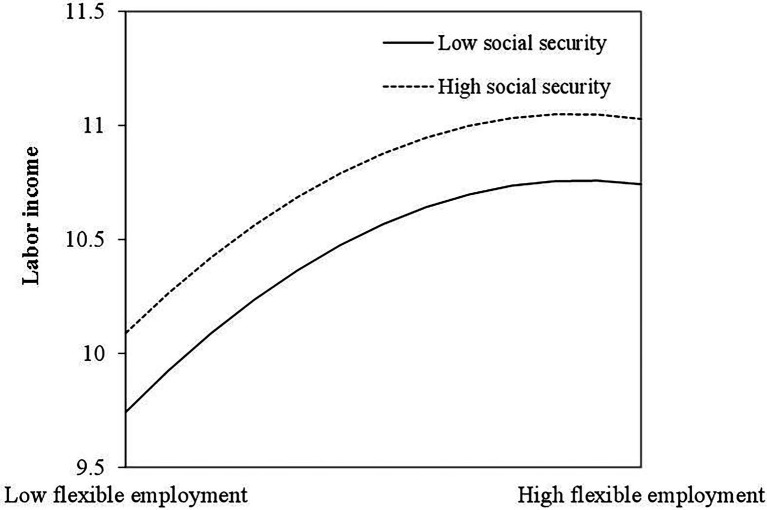
The indirect moderating effect of social security.

Similarly, we got similar results in the moderating test of social insurance on labor income. In Model 3 of Table 3, social security positively predict labor income (*b* = .125, *p* < .001), but the value of the interaction item of FE and SI is not significant. Figure 3 shows the indirect moderating effect. The inverted U-shaped relationship between FE and LI do not change at both high and low level of social insurance. However, it can be seen that employees gained more labor income at high level of social insurance than that at low level, indicating an enlightening discovery for practice.

#### Test of Mediated Moderating Effect

In the intermediary adjustment model shown in Model 7 of Table 3, social insurance has a positive significantly effect on workers’ wellbeing (*b* = .057, *p* < .001). Bootstrap test is performed on the intermediary adjustment model, and the results are shown in Table 4. Under different social security levels, the indirect effects of flexible employment on workers’ wellbeing through labor income are significantly negative (the 95% CI interval does not include 0 in the three levels), indicating that the moderating effect of social security is significant. Hypothesis 3b is supported.

**Table 4:** Bootstrap test results of moderated mediating effect.

Moderated variable	Level	Effect	SE	95%CI
Social insurance	Low(−1 SD)	.0059	.0016	[.0031, .0094]
	Medium	.0057	.0014	[.0033, .0088]
	High(+1 SD)	.0055	.0017	[.0028, .0098]

## Discussion

### Conclusion

This study uses 5,439 observations in the 2017 CGSS data to explore the relationship between flexible employment and workers’ wellbeing and analyzes the mediating role of labor income and the moderating role of social security, which draws the following conclusions:

There is an inverted U-shaped relationship between flexible employment and workers’ wellbeing, that is, flexible employment can enhance workers’ wellbeing within a certain range, and then reduces the level of wellbeing after reaching the peak. According to the analysis results, appropriate flexibility, providing employees with freedom to work, can improve workers’ wellbeing and labor income in varying degrees. However, with the continuous development of this state, flexibility may evolve to the opposite, manifesting as “extremely unstable.” This negative impact will have a greater impact on individuals. In other words, flexibility and autonomy cannot completely compensate for “informality,” which results in the loss of workers’ wellbeing owing to insecurity and instability. The risk of unemployed at any time and the sense of insecurity felt at work have a direct negative impact on the happiness and health status of employees. These workers who lack stable jobs and economic security are often viewed as typical “urban drifters” with a low sense of social belonging. Thus, it is hard for them to be embraced by strong sense of happiness.

Labor income mediated the relationship between flexible employment and workers’ wellbeing. Flexible employment not only directly affects the wellbeing of workers, but indirectly affects that through labor income. Thus, inadequacy in satisfaction of labor income may be one of the explanatory mechanisms for why flexible employment are more likely to develop low-level wellbeing. The effect of flexible employment on labor income is basically the same as the effect of flexible employment on happiness. Flexible employment can help increase the income of workers within a certain range, but this “good” situation will not increase with the increase of flexibility. However, continuing to rise, it reduces labor income after a certain level. There are two possible explanations for flexible employment to reduce labor income. One is, higher-income groups (such as white-collar workers and intellectuals) deliberately sacrifice their labor income in order to seek flexibility and autonomy of work to obtain more disposable time; the other is, the instability of flexible employment itself is directly lead to the instability of labor income, which in turn affects its total income level.

Our findings confirmed the moderating role of social security in the mediating effect of labor income. Increasing the level of social security can weaken the negative impact of flexible employment on workers’ wellbeing, or even turn it into a positive impact. This effect is not significant. Furthermore, this result shows that social security can comfort and compensate the unhappiness of flexible employees to a certain extent, but only increasing the level of social security cannot significantly improve their happiness. This is related to a wide range of factors affecting happiness. Although the overall level of labor income is higher than the low-level social security group, the income of flexible employees in high-level social security suffers more “income discount” because of the high cost of social insurance. Those who engaged in flexible employment may have the possibility of paying social insurance at their own expense, which will affect their incomes. As we all known, the compensatory effect of pension works only after retirement for individuals, and medical insurance provide medical expenses compensation only after disease problems emerging.

### Research Contribution

#### Theoretical Implications

The most substantial contribution of this study is the examination of a mediated moderation model which embraces four variables to explore the inner mechanism of flexible employment’s influence on happiness. As far as we are aware, the existing literature on flexible employment mainly involves the legal risks of labor relations and pay less attention to employees’ wellbeing. Although some scholars have studied that through relevant data, they do not take income and social security into consideration. This study examines both linear and non-linear effects of flexible employment on happiness and simultaneously incorporates labor income and social security into the research model as mediating and moderating variables to interpretate how flexible employment affects workers’ wellbeing, revealing that labor income is one of the primary mediation mechanisms and the level of social security is one way to account for the heterogeneity in the impact of flexible employment on workers’ wellbeing.

In addition, the study redefines “flexible employment” from the perspective of “flexibility” and enriches the research perspective. In the past, most researches used “informal employment” rather than “flexible employment” to explore. Those definitions tend to focus on “informal.” When selecting research objects for group restraint, more often they conduct it based on whether employees signed a labor contract and whether they paid social insurance. This research measures “flexible employment” from three aspects, including signation of labor contract, full-time or part-time work, and one or multiple part-time jobs. Considering the attribute of “flexibility and autonomy,” we take both informal employees and formal employees with highly flexibility into this model. Apart from this, non-linear analysis provides new evidence for the study of the relationship between flexible employment and workers’ wellbeing from a new perspective.

#### Practical Implications

First, Improving the quality of flexible jobs is a top priority. The impact of flexible employment on labor income is closely related to the quality of employment. High employment quality can increase income and increase the sense of job satisfaction. It can also maximize the time advantages and skill benefits of employees to narrow the income gap between flexible and formal employees. This will enhance the happiness of workers. The quality of employment should also include protecting the basic rights and welfare of workers as much as possible and meeting their safety needs. When income levels no longer cause too much “damage” to happiness, the flexible and autonomous advantages of flexible employment can play a greater role and even produce higher life satisfaction than formal employment. The inverted U-shaped relationship between flexible employment and labor income and happiness can also be effectively improved. In addition, from the perspective of social development, high-quality flexible employment can increase labor productivity and promote economic transformation, which is also of great significance to high-quality development.

Second, moderately reducing social insurance rates is a beneficial policy for flexible employees. The high rate of social insurance not only easily increases the probability of informal employment of enterprises, but reduces the willingness of flexible employees to voluntarily pay for social insurance. Appropriate reduction of social insurance rates can improve the level of protection for flexible employees, increase social insurance coverage, and reduce the risk of flexible employees becoming poor due to illness. At the same time, pension income can narrow the income gap (Yu et al., 2021), which is conducive to magnifying or enhancing the upward part of the curve of “an U-shaped relationship between flexible employment and workers’ wellbeing under high social security,” thereby enhancing the sense of life security and happiness. Although the state has paid attention to the social security issues of flexible employment groups and adopted multiple payment standards for flexible employees to choose by themselves, some favorable policies have not yet benefited this group, such as some social security fee reduction measures issued during the epidemic. Therefore, the social security system for flexible employees needs to be continuously improved. Of course, in addition to social security, other factors that affect the happiness of workers should also be paid attention to.

### Limitations

Several limitations must be considered when interpreting the results of the present study. Firstly, the data adopts the cross-sectional data of 2017, but flexible employment has been in a process of dynamic change in recent years. The impact of flexible employment on workers’ wellbeing may vary across time nodes. In future research, the data of multiple periods can be considered for longitudinal comparative research to explore the change trend of the impact of flexible employment on workers’ wellbeing; Secondly, flexible employment has a wide range of forms where different types of flexible work have different effects on workers’ wellbeing. Future research can classify and distinguish industries or work contents to further study this theme; Finally, there are many paths for the impact of flexible employment on workers’ wellbeing, and the impact results under different paths may be different. The analysis of other path factors is also worth exploring.

## Data Availability Statement

The original contributions presented in the study are included in the article/supplementary material, and further inquiries can be directed to the corresponding author.

## Author Contributions

DJ: conceptualization, methodology, and project administration. TL and QL: data curation, formal analysis, writing—original draft, and writing—review and editing. All authors contributed to the article and approved the submitted version.

## Funding

This research was funded by General Research Project of Humanities and Social Sciences of the Ministry of Education, grant no. 20YJCZH209.

## Conflict of Interest

The authors declare that the research was conducted in the absence of any commercial or financial relationships that could be construed as a potential conflict of interest.

## Publisher’s Note

All claims expressed in this article are solely those of the authors and do not necessarily represent those of their affiliated organizations, or those of the publisher, the editors and the reviewers. Any product that may be evaluated in this article, or claim that may be made by its manufacturer, is not guaranteed or endorsed by the publisher.
